# Seasonal freeze-thaw drives arsenic metabolism dynamics in sediments of a cold-region lake: microbial gene and community responses

**DOI:** 10.1128/aem.00485-26

**Published:** 2026-05-05

**Authors:** Wenjing Shi, Haoran Xu, Weiping Li, Jin Xu, Yan Qin, Long Bai

**Affiliations:** 1 School of Energy and Environment, Inner Mongolia University of Science and Technology 177540 , Baotou, China; 2 Collaborative Innovation Center for Ecological Protection and Comprehensive Utilization of the Inner Mongolia Section of the Yellow River Basin, Inner Mongolia University of Science and Technology 177540 , Baotou, China; University of Milano-Bicocca, Milano, Italy

**Keywords:** seasonal freeze-thaw, As metabolic genes, As-metabolizing microbial community, cold-region lake

## Abstract

Seasonal freeze-thaw process critically regulates arsenic (As)-metabolizing microorganisms (AMMs) in lake sediments through redox-nutrient dynamics. However, the current understanding of microbially mediated As transformation and mobilization processes under freeze-thaw dynamics remains poorly constrained. This study systematically investigated the successional patterns and driving mechanisms of As-metabolizing functional genes and microbial community structures of AMMs in lake sediments during the freeze-thaw process. This study focused on Lake Wuliangsuhai—a typical mid-high latitude lake in northern China—and employed an integrated framework of co-occurrence networks, Mantel tests, and correlation heatmaps. The results revealed that freeze-thaw processes drive significant AMMs restructuring, altering taxonomic composition and functional gene expression while controlling As environmental fate through regulating microbial metabolic functions, altering redox regimes, and restructuring community interaction networks. Co-occurrence network revealed stage-specific restructuring of microbial interactions during freeze-thaw process: robust mutualism established foundational networks during the pre-freezing stage; simplified modularity reflected functional differentiation during the ice-covered period; post-thaw modularity increased during structural reorganization; and synergistic complexity characterized adaptive strategies during the open-water period. Integrated results from Mantel tests and correlation heatmaps identified total nitrogen (TN), total phosphorus (TP), Fe(II), As(III), and As(V) as key succession drivers, with stage-dependent influences on AMMs. This work elucidates fundamental regulatory mechanisms through which seasonal freeze-thaw processes govern As-metabolizing gene dynamics and microbial ecological functions in lake sediments. It further highlights how microbially driven As transformations exacerbate sedimentary contamination risks under climate change, providing critical theoretical foundations for regional water management and pollution mitigation strategies.

**IMPORTANCE:**

Seasonal freeze-thaw processes in cold lakes dramatically control arsenic pollution risks, but how microbes drive this process remains a critical knowledge gap. This study reveals how winter ice cover and spring thaw create “hot moments” for toxic arsenic release by activating specialized sediment microbes, necessitating stage-specific water quality management. Crucially, nutrient loading (total nitrogen/total phosphorus) exacerbates arsenic (As) transformations by stimulating functional gene expression and microbial interactions. As climate change shortens ice seasons, these contamination pulses may become more frequent and severe. By identifying key microbial indicators and high-risk transition periods, our findings empower lake managers to predict arsenic hazards. This science is vital for safeguarding freshwater ecosystems and human health across ice-affected regions worldwide.

## INTRODUCTION

Arsenic (As), a widely distributed toxic metalloid, ranks among the top 10 chemicals of major public health concern according to the World Health Organization (WHO) (1). Arsenite (As(III)) exhibits greater mobility and toxicity than arsenate (As(V)) (2), with its toxicity potentially reaching 60 times that of As(V) (3). Globally, an estimated 140 million people are exposed to drinking water with As concentrations exceeding the WHO guideline limit of 10 μg/L (4), directly endangering human health. In lacustrine systems, sediments represent a critical reservoir for As (5), functioning not only as a long-term sink but also as a dynamic, biologically active interface that regulates its speciation and mobility. Changes in environmental conditions can transform sediments into a secondary source, leading to As remobilization and secondary water contamination.

The mobility and transformation of As in sediments are regulated by multiple physicochemical factors, including redox conditions, pH, and binding modes with iron (hydro)oxides (6, 7). However, increasing evidence indicates that microbially mediated metabolic cycling plays a central role in controlling As speciation, mobility, and toxicity (8–10). Microorganisms drive key transformations through a series of functional genes (such as *aioA, arsC, arrA,* and *arsM*), including oxidation, reduction, methylation/demethylation, and transport (11–15). These processes are often tightly coupled to the biogeochemical cycles of iron, sulfur, carbon, and nutrients. Consequently, understanding As dynamics at the sediment-water interface requires a direct investigation of these microbial functional potentials, as water-column measurements alone cannot resolve the underlying mechanisms.

Despite advances in understanding As cycling in estuarine and marine systems (16–18), significant gaps remain regarding these processes in mid- to high-latitude lakes subjected to seasonal freeze-thaw cycles. This understanding becomes particularly urgent for lakes in cold regions subjected to pronounced seasonal freeze-thaw cycles. The annual progression from open water to ice cover and back to thaw induces abrupt, drastic shifts in critical sediment conditions: temperature plunges, oxygen transport is severely restricted, and redox gradients are reconfigured. These physicochemical transitions can create transient hot moments of microbial metabolic activity, potentially amplifying the rates of As transformation and mobilization during key seasonal windows (17–19). The microbial actors driving these transformations possess a suite of functional genes that orchestrate As redox cycling. As metabolism encompasses diverse processes, including oxidation, reduction, methylation/demethylation, and transport/transformation, mediated by multiple functional genes. Microbial-driven As speciation transformations correlate with the abundance and potential activity of functional genes such as *aioA, arsC, arsM*, and *arsI* (11, 15). Seasonal succession of microbial communities may regulate the abundance patterns of these genes and associated functional guilds, thereby affecting As mobility, toxicity, and ecological risk (11). For example, As-metabolizing microorganisms can oxidize the more toxic and mobile As(III) to As(V), which is generally less mobile and more readily adsorbed, reducing bioavailability; conversely, arsenate respiration and cytoplasmic reduction can facilitate the release of solid-phase As into porewater and overlying water (8, 20–22). These opposing microbial processes collectively determine the direction and magnitude of As cycling under shifting redox conditions. Seasonal succession of microbial communities and genes may determine the net direction of As fixation or release at the sediment-water interface. However, the microbial genetic machinery and community responses underpinning these hot moments in seasonally frozen lake sediments are poorly characterized, representing a critical knowledge gap for predicting As behavior in these vulnerable ecosystems.

Wuliangsuhai (WLSH) Lake, a large shallow freshwater lake on the Hetao Plain of northern China, serves as an ideal model system to address this gap. It experiences an annual ice-covered period of approximately 5 months (November–April). During spring thawing, pronounced fluctuations in redox gradients develop at the sediment-water interface, and rapid temperature increases combined with ice melt-induced sediment resuspension create activating conditions for sediment microbial metabolism. The surrounding Hetao Plain is a known high-As region, and WLSH receives agricultural drainage, leading to concerns over As accumulation (16). During the prolonged ice-covered period, the lake remains under reducing conditions, promoting the accumulation of As in reduced forms and sediment-bound states associated with iron (hydro)oxides (23). The seasonal shift from ice-bound, reducing conditions to a dynamic, oxic-post-thaw environment likely triggers profound changes in the sediment microbial community and its functional potential for As metabolism, creating a pulse risk for As release.

Accordingly, this study focuses on As-metabolizing microorganisms (AMMs) harboring As metabolic cycling genes, investigating the spatiotemporal dynamics, interaction networks, and environmental regulators of As metabolic functional genes and microbial taxa in WLSH lake sediments across a seasonal freeze-thaw cycle. Specifically, this work aims to (i) characterize differential responses of As metabolic genes and microbial community structures to freeze-thaw processes; (ii) elucidate relationships between As metabolic genes/microbial communities and environmental factors during freeze-thaw transitions; and (iii) identify key functional genes and AMMs that play dominant roles in regulating As biogeochemical cycling in seasonally frozen lake sediments.

## MATERIALS AND METHODS

### Study area and sample collection

WLSH Lake (40°36'–41°03'N, 108°43'–108°57'E) is situated in Urad Front Banner, Bayannur City, Inner Mongolia Autonomous Region, China. It is the largest freshwater lake in the Yellow River Basin and the largest wetland at the same latitude globally (24). The lake covers an area of 325.31 km², with depths ranging from 0.5 to 3 meters and an average depth of approximately 0.7 meters (25). Located in the cold-arid region of the Mongolian-Xinjiang Plateau, this representative lake in Inner Mongolia’s Yellow River section experiences an annual ice-bound period of 5 months. The main inflow occurs in the northern and western parts of the lake, while the outflow is located in the southern section (25, 26). As the main recipient of agricultural effluent from the Hetao Irrigation District, its dominant water source, WLSH, is prone to As contamination and enrichment. Studies have shown that As enrichment was found in WLSH, and the surface sediment is mostly polluted during the ice-bound period; the potential ecological risk of As cannot be ignored in WLSH during the ice-bound period (27).

**Fig 1:**
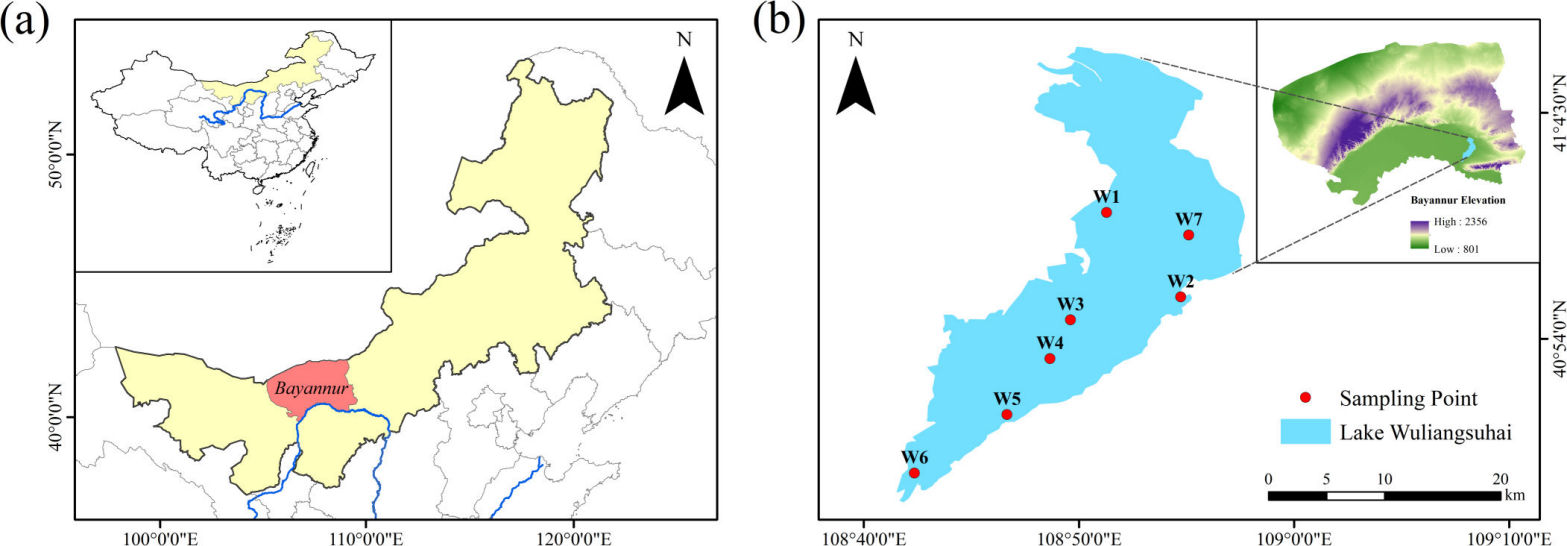
Sampling site distribution map. (**a**) Overview map of the study region. (**b**) Detailed map of sampling points in the Bayannur–Lake Wuliangshai area.

Sediment samples were collected from the study area during four critical hydrological periods: pre-freeze period (October 2023), ice-covered period (January 2024), post-thaw period (April 2024), and open-water period (August 2024). Based on field investigations and considering the lake’s hydrological pattern and regional hydrographic features, seven representative sampling sites (W1–W7) were established (Fig. 1). W1 is the main inlet of the total drainage channel. W2 is a reed-dominated area with abundant emergent macrophytes but lacking submerged vegetation. W3 is situated in the central part of the lake, surrounded by reeds and featuring submerged vegetation beneath the water surface. W4 and W5 are located in the open-water region in the south (tourism area), both with submerged aquatic vegetation. W6 lies near the drainage outlet, serving as the lake’s outflow point, while W7 is in the northern open-water area. All sites were georeferenced using GPS. Sediment samples were collected using a Swedaq KC mod B undisturbed corer (28), with the sampling depth adjusted according to the lake depth. To ensure analytical accuracy and reliability while minimizing experimental error, three parallel sediment cores were collected at each site within an approximate 1-meter radius. Then, visible plant debris and macrofauna were removed. The sediment from each core was then thoroughly homogenized to form a composite replicate sample. Each homogenized sample was subsequently divided into two portions and stored in sterilized sealed bags. One portion was stored on dry ice for metagenomic analysis. The other portion was kept at low temperature and protected from light and transported back to the laboratory for the determination of total arsenic (TAs), total nitrogen (TN), total phosphorus (TP), and other relevant sediment parameters.

This study involved the measurement of As(III), As(V), total As, Fe(II), total Fe, total nitrogen (TN), total phosphorus (TP), pH, and organic matter (OM) concentrations in the sediments, following the analytical methods detailed in a study by Shi et al. (22). To avoid potential contamination during the experimental procedures, only glassware was used throughout the experiment. All glassware was soaked in 1:3 (vol/vol) nitric acid for more than 24 h, followed by thorough rinsing with ultrapure water and oven-drying. All reagents used were of analytical grade or higher, and the water used was ultrapure water (Milli-Q, Millipore, ≥18.2 MΩ·cm). To ensure the accuracy of experimental results, blank controls and parallel samples were included in the analysis. The standard deviation of all analytical methods was within 5%.

### Metagenomic sequencing and data analysis

A total of 28 sediment samples were subjected to metagenomic sequencing. Genomic DNA was extracted from sediment samples using the E.Z.N.A. Soil DNA Kit (Omega Bio-tek, USA). DNA concentration, purity, and integrity were assessed, and qualified DNA was fragmented to an average size of 350 bp using a Covaris M220 (China Gene Company, Ltd.) for paired-end library construction. Sequencing was performed on an Illumina NovaSeq 6000 platform by Majorbio Bio-Pharm Technology Co., Ltd (Shanghai, China). After quality control using Fastp, which included adapter trimming and removal of low-quality reads (length < 50 bp, quality score < 20, or containing ambiguous bases), a total of approximately 2,512.8 million high-quality paired-end reads were obtained, with an average of 89.7 ± 5.9 million reads per sample, corresponding to an effective sequencing depth of 12 Gb. Detailed per-sample sequencing statistics are provided in File S1. For details on periodic coding, quality control data, and statistical information, please refer to the metadata file included in File S1.

Raw reads were quality-filtered and assembled *de novo* using MEGAHIT (29), and contigs with lengths ≥ 350 bp were retained. Open reading frames (ORFs) were predicted from the contigs using MetaGene (30). Predicted genes (nucleotide lengths ≥ 100 bp) were translated and clustered with CD-HIT (31) at 90% identity and 90% coverage to generate a non-redundant gene catalog. The longest sequence within each cluster served as the representative gene. Gene abundance in each sample was quantified by aligning the high-quality reads back to this non-redundant catalog using SOAPaligner (32), with a 95% identity threshold.

Microbial taxonomic classification was performed using predicted ORFs from the metagenomic assemblies. ORFs were annotated against the NCBI non-redundant (NR) database using DIAMOND (v2.0.13) (33), with an e-value cutoff of 1e^–5^. Taxonomic assignment from the phylum to the genus level was performed using the lowest common ancestor (LCA) algorithm implemented in the Majorbio Cloud Platform (https://cloud.majorbio.com), applying standard identity thresholds (≥60% for phylum and ≥95% for genus). For functional annotation, genes were aligned against the Kyoto Encyclopedia of Genes and Genomes (KEGG) database (https://www.genome.jp/kegg/) using DIAMOND (e-value ≤ 1e^–5^). KEGG Orthology (KO) identifiers were used to classify the genes involved in As metabolic pathways.

To identify As metabolism genes, this study screened the metagenomic KO annotations for 22 As cycling genes (with corresponding KO identifiers) compiled from prior studies and categorized them into four functional groups (oxidation, reduction, methylation/demethylation, and transport/transformation) (34). These genes were classified into four functional categories according to their roles in sediment As metabolic pathways. The KO identifiers, gene names, functions, and classifications related to sediment As metabolism were provided in Table S2. AMMs were defined as microbial taxa (archaea, bacteria, and fungi) that harbor at least one of these As cycling genes. In practice, AMMs were identified by extracting the taxa linked to the As gene set from the total community, based on the gene-taxonomy associations established during the metagenomic annotation process. All subsequent community composition, diversity, and network analyses were conducted on this AMM subset. In this study, a total of 140 archaeal genera, 2,273 bacterial genera, and 7 fungal genera were identified as AMMs in the WLSH sediment samples.

### Data statistics and analysis

Raw data of environmental physicochemical measurements were initially organized and pre-processed using Microsoft Office Excel 2024 (Microsoft, USA). Statistical analyses were conducted using SPSS 26.0 (IBM, USA). Prior to statistical analyses, the normality of all data was formally evaluated using the Shapiro-Wilk test in SPSS 26.0. Variables that did not satisfy the normality assumption (*P* < 0.05) were log-transformed to approximate a normal distribution. Where transformed data still deviated significantly from normality, corresponding non-parametric tests (specifically, the Kruskal-Wallis test) were applied for subsequent analysis. For multivariate and correlation-based analyses, variables were z-score standardized (mean-centered and scaled to unit variance) to ensure comparability among parameters measured in different units.

Microbial alpha diversity indices (ACE, Chao, Shannon, and Simpson) of AMMs were calculated on the Majorbio Cloud Platform (https://cloud.majorbio.com). Community structure differences among sediment samples were visualized using non-metric multidimensional scaling (NMDS) based on Bray-Curtis distances. Significant differences in community composition and taxon abundance among groups were tested using the Kruskal-Wallis rank-sum test, followed by false discovery rate (FDR) correction and Tukey-Kramer post hoc tests for multiple comparisons.

Associations between the concentrations of As, iron (Fe), and phosphorus (P) during the freeze-thaw cycle and the abundance of dominant functional genes and microbial taxa were examined using Spearman correlation analysis. Mantel tests were performed in R software (version 3.6.1) to evaluate significant associations between dominant bacteria and As-Fe-P dynamics/environmental factors in WLSH sediments. Co-occurrence network analysis was conducted using Gephi 0.10.1 to explore interactions among AMMs (35). Furthermore, the key drivers of the relative abundance of keystone AMMs and dominant As cycle genes were identified using random forest analysis implemented with the “randomForest” package (36).

Microbiological statistical analysis and visualization were performed on the Majorbio cloud platform (https://cloud.majorbio.com). Figures were prepared using OriginPro 2024 (OriginLab, USA) and ArcGIS Desktop 10.8 (Environmental Systems Research Institute, USA).

## RESULTS

### As metabolic genes abundance in sediments during the freeze-thaw process

**Fig 2:**
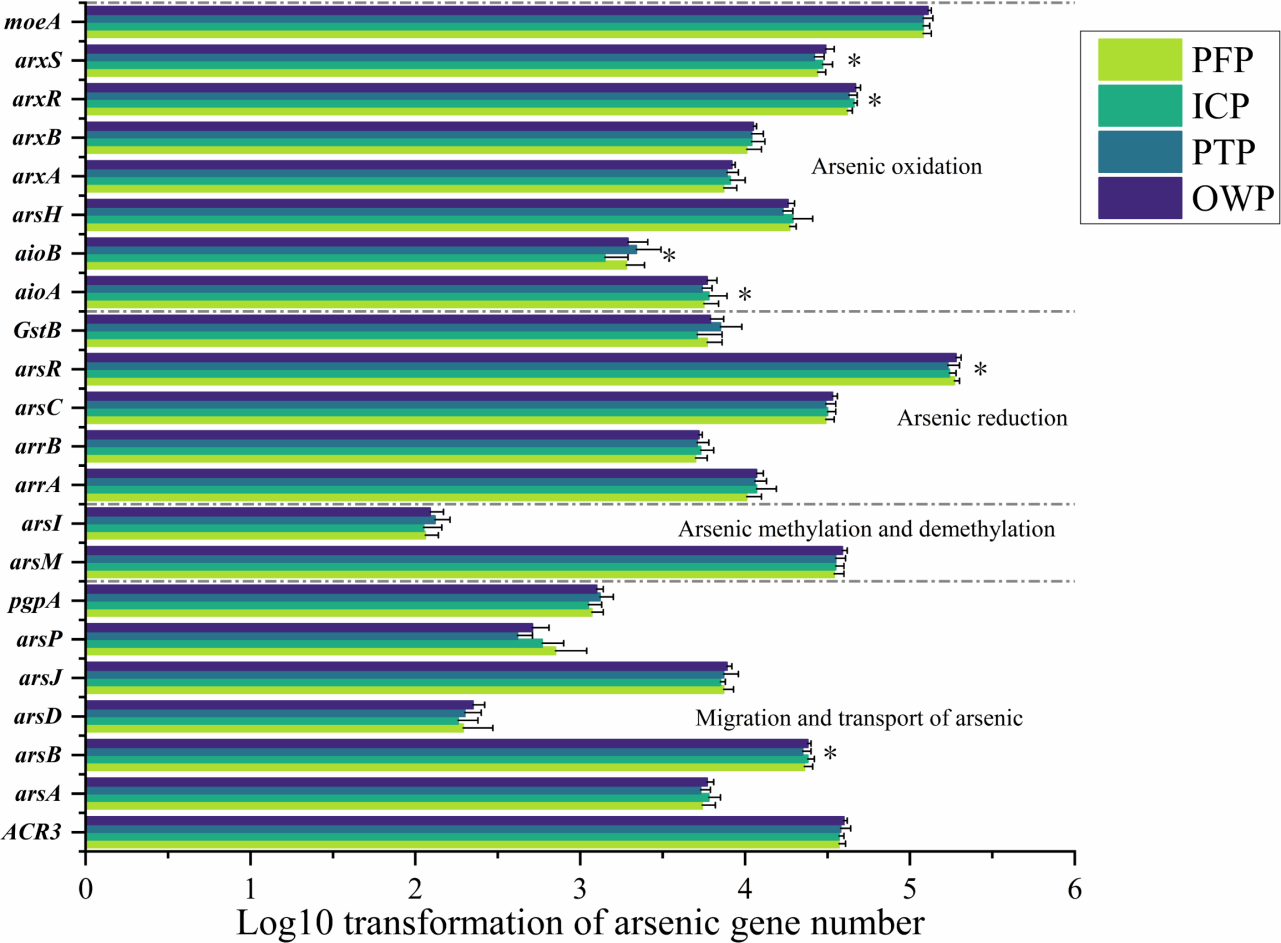
Abundance of As cycle genes in sediments of WLSH during the freeze-thaw process. The asterisks (*) indicate the significant (*P* < 0.05) differences among the four periods. PFP represents pre-freeze period, ICP represents ice-covered period, PTP represents post-thaw period, and OWP represents open-water period.

As cycling genes representing four functional modules, As oxidation, As reduction, As methylation/demethylation, and As migration/transport, were detected in WLSH sediments across all freeze-thaw periods (PFP, ICP, PTP, and OWP) (Fig. 2). While the overall functional profile remained broadly stable throughout the seasonal cycle, several module-specific marker genes showed clear period dependence, with the most evident contrasts involving the ice-covered period and the ice-free stages (PTP/OWP).

Within the oxidation module, the abundances of *aioA* and *aioB* differed significantly among periods (*P* < 0.05). *aioB* abundance was lowest during the ice-covered period and increased progressively during the ice-free period; although present at lower absolute abundance, *aioA* also showed statistically significant temporal variation. The regulatory genes of the *arx* system (*arxR* and *arxS*) similarly displayed significant differences (*P* < 0.05), peaking during the open-water period, whereas the *arxA/arxB* ratio remained relatively stable. In the reduction module, As reduction/resistance genes remained consistently abundant; *arsR* exhibited a significant periodicity effect (*P* < 0.05), reaching its highest abundance during the open-water period, while *arrA, arrB,* and *arsC* exhibited relatively minor fluctuations across periods. For the methylation/demethylation module, *arsM* remained consistently abundant across all periods, whereas *arsI* was detected at lower levels with a slight increase during the post-thaw period. Within the migration/transport module, genes encoding transporters and efflux pumps were widely detected. *arsB* abundance varied significantly among periods (*P* < 0.05), being higher during ice-covered and open-water periods than in the post-thaw period, while other transport genes showed less seasonal variation.

### Community composition of AMMs in sediments during the freeze-thaw process

**Fig 3:**
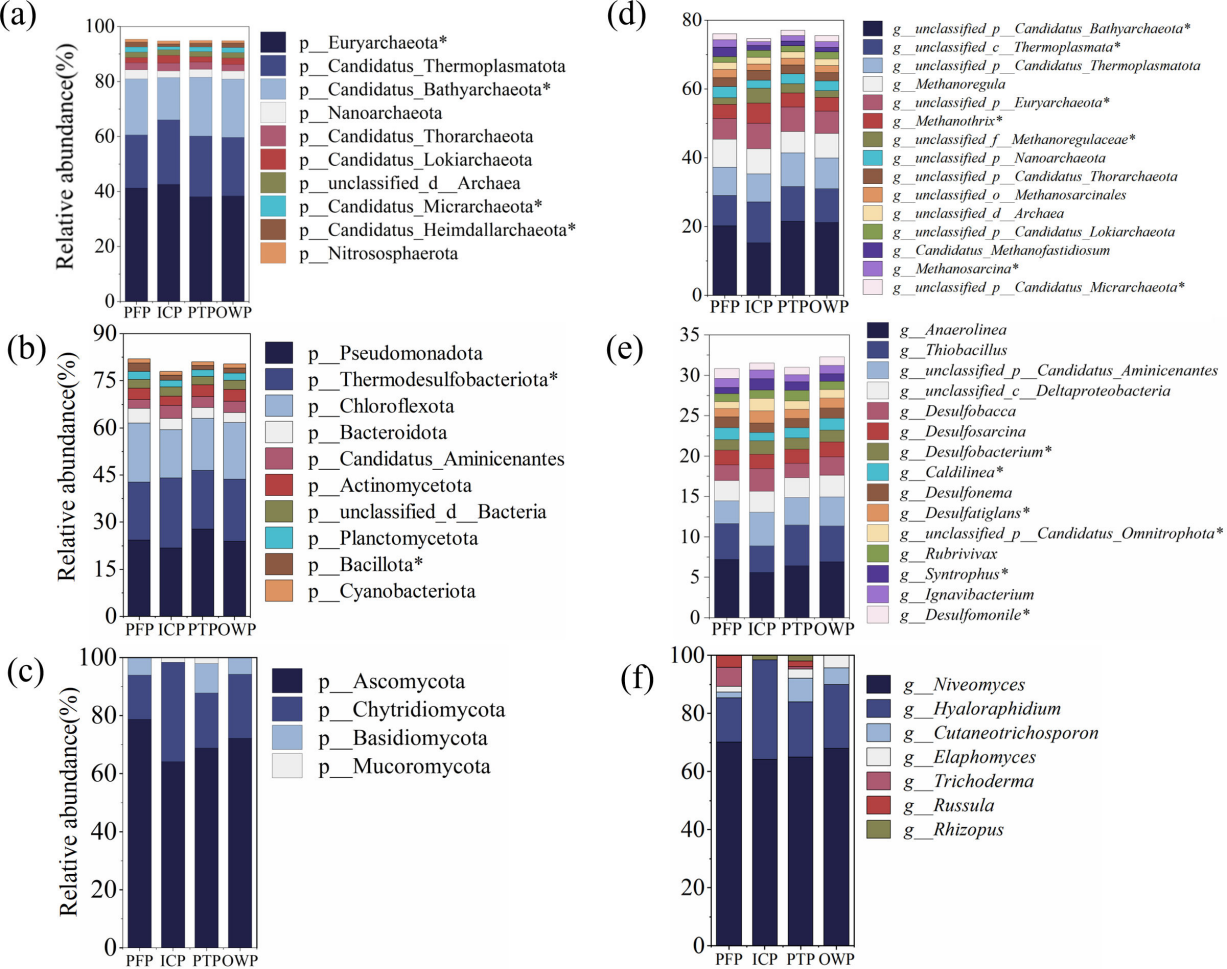
The AMMs communities at the (**a–c**) phylum (top 10) and (**d–f**) genus (top 15) levels during freeze-thaw periods. (**a**) and (**d**) are archaea, (**b**) and (**e**) are bacteria, (**c**) and (**f**) are fungi. The asterisk (*) indicates the significant differences (*P*  <  0.05) among the four periods. PFP represents pre-freeze period, ICP represents ice-covered period, PTP represents post-thaw period, and OWP represents open-water period.

Metagenomic sequencing revealed As-metabolizing microorganisms in WLSH sediments spanning 28 archaeal phyla, 140 bacterial phyla, and 4 fungal phyla, comprising 138 archaeal genera, 2,273 bacterial genera, and 7 fungal genera (details shown in SM). At the phylum level, archaeal AMMs (Fig. 3a) were dominated by Euryarchaeota and Candidatus Bathyarchaeota, whose relative abundances exhibited significant temporal variation among the four periods (*P* < 0.05). In particular, Euryarchaeota showed a marked increase during the ice-covered period, whereas several minor archaeal phyla displayed reduced contributions. Bacterial AMMs (Fig. 3b) were primarily affiliated with Pseudomonadota, Chloroflexota, and Bacteroidota. Significant differences were detected for multiple bacterial phyla, including Thermodesulfobacteriota and Bacillota (*P* < 0.05), indicating sensitivity of bacterial assemblages to freeze-thaw dynamics. Fungal AMMs (Fig. 3c) were consistently dominated by Ascomycota, with significant but comparatively smaller fluctuations among periods.

At the genus level, clearer succession patterns emerged (Fig. 3d through f). Among archaea (Fig. 3d), methanogenic genera such as *Methanothrix* showed significantly higher relative abundances during the ice-covered period (*P* < 0.05), while several unclassified archaeal genera also exhibited period-specific increases. For bacterial AMMs (Fig. 3e), sulfate-reducing genera including *Desulfobacterium*, *Desulfatiglans*, and *Desulfomonile* were significantly more abundant during the ice-covered period (*P* < 0.05). In contrast, genera such as *Anaerolinea* and *Thiobacillus* displayed significantly higher abundances during the pre-freeze and open-water periods, with pronounced declines under ice-covered conditions (*P* < 0.05). Fungal genera (Fig. 3f), dominated by *Niveomyces* and *Hypholoma*, also showed significant but less pronounced temporal variations.

These results demonstrated that the freeze-thaw process strongly restructured AMMs community composition, with the ice-covered period exerting the most distinct selective pressure.

### Comparison of sediment As metabolism genes and AMM communities across freeze-thaw periods

NMDS analysis revealed distinct clustering patterns of sediment samples in the ordination space, indicating that both As metabolism genes (Fig. S1a) and AMMs community structures (Fig. S1b) varied across the seasonal freeze-thaw process. This demonstrated that the freeze-thaw process of the lake significantly altered the composition of sedimentary As metabolism genes and AMMs communities (*P* < 0.05). However, alpha diversity indices, including Chao, ACE, Shannon, and Simpson (Fig. S2), showed no significant differences throughout the entire freeze-thaw periods (*P* > 0.05), implying a potential stability of the As-metabolizing microbial communities in lake sediments. This stability may be attributed to the intrinsic microbial stress resistance mechanisms, and the possibility that the freeze-thaw dynamics do not exceed critical thresholds required to disrupt the microbial-mediated As biogeochemical cycling. These findings indicate that despite seasonal freeze-thaw stress, As-metabolizing microorganisms in cold-region lakes maintain ecological functionality through genomic and metabolic adaptations.

### Co-occurrence networks of As metabolic genes and AMMs during the freeze-thaw process

**Fig 4:**
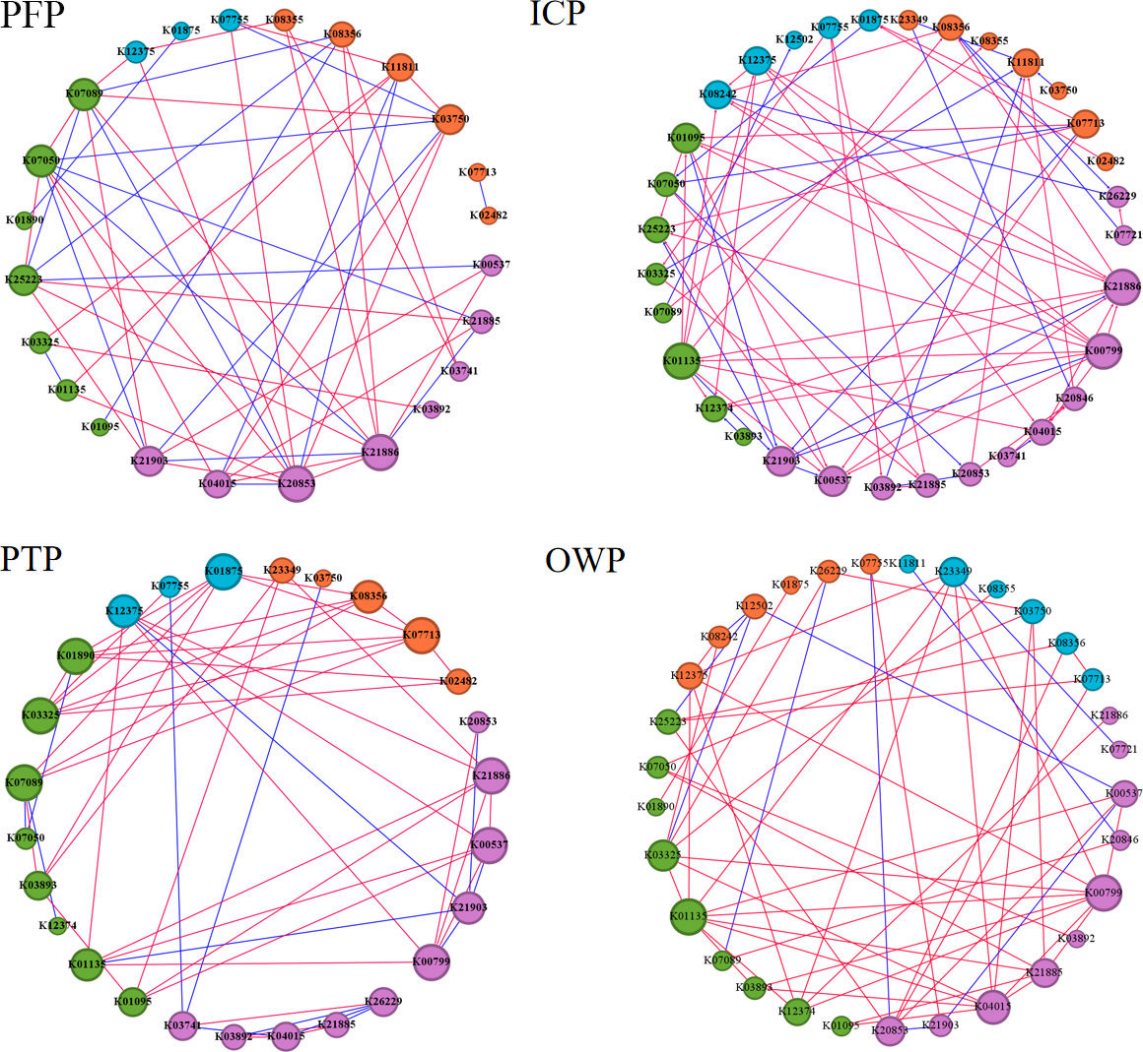
Occurrence network analysis revealed genes involved in the sediment As cycle during freeze-thaw processes. The node size shows the intensity of the links with other nodes (genes). Nodes with purple, green, orange, and blue colors represent the genes involved in As reduction, migration and transport of As, As oxidation, and As methylation and demethylation, respectively. The colored line shows the correlation of the two nodes, with the red line for the positive relationship and the blue line for the negative relationship. The basic topological properties of these networks are shown in Table S4. PFP represents pre-freeze period, ICP represents ice-covered period, PTP represents post-thaw period, and OWP represents open-water period.

Topological characteristics of the co-occurrence network of As metabolic genes revealed that seasonal freeze-thaw dynamics significantly restructured interaction networks and stability patterns among As metabolic genes. The network constructed for the pre-freeze period exhibited the most compact structure, with pronounced synergistic interactions among genes, indicating strong functional linkage within the system. During the ice-covered period, peak node/edge counts and elevated modularity (Fig. 4; Table S4) demonstrate the formation of tightly integrated functional modules under hypoxic/low-temperature stress to enhance environmental adaptation. In the post-thaw period, further increases in modularity and functional compartmentalization imply ongoing network reconstruction and functional adjustment. Conversely, during the open-water period, a decentralized structure with the highest positive correlation ratio (Table S4) reflects optimized synergistic interactions for efficient metabolic processes among As metabolic genes at this stage. Collectively, the topological structure of the As metabolic gene co-occurrence network in sediments showed a significant dynamic succession with seasonal freeze-thaw periods, reflecting the reshaping process of functional gene expression and its collaborative network by environmental fluctuations, which, in turn, affected the migration and transformation of As in the sedimentary environment and ecological risks.

**Fig 5:**
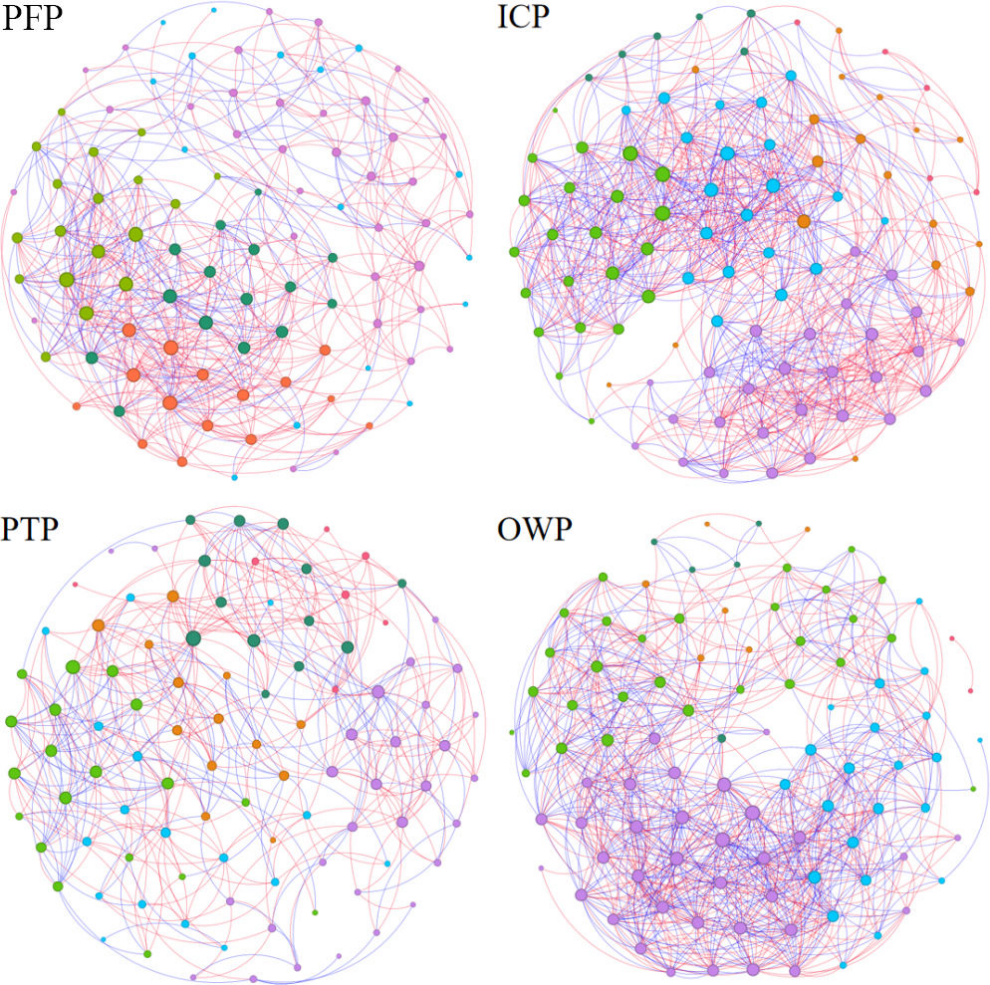
Occurrence network analysis of AMMs involved in sedimentary As cycling during different periods. Nodes in different colors represent different modules, and nodes with the same color belong to the same module but indicate different AMMs. Node size reflects the strength of connectivity with other nodes. Colored edges between nodes represent correlations: red lines indicate positive correlations, while blue lines indicate negative correlations. The basic topological properties of these networks are summarized in Table S5. PFP represents pre-freeze period, ICP represents ice-covered period, PTP represents post-thaw period, and OWP represents open-water period.

Co-occurrence network analysis of AMMs revealed decreased topological parameters (edge count, average degree, and graph density) during the post-thaw period (Fig. 5; Table S5), indicating reduced connectivity within the microbial community structure. This decline in network complexity and stability may be attributed to enhanced environmental perturbations post-thaw, rapid nutrient flux alterations, and allochthonous species inputs following lake thaw. These factors likely intensified niche differentiation among sediment microbes, reducing the necessity for interspecies interactions and weakening overall microbial associations. During the seasonal freeze-thaw processes, the AMM networks in the pre-freeze, ice-covered, and post-thaw periods were dominated by positive (mutually facilitative) interactions over competitive ones, suggesting that under cold and nutrient-limited conditions, microbial communities enhance their stability and ecological resilience through synergistic metabolism and ecological complementarity. Conversely, the open-water period network showed nearly equal proportions of positive and negative correlations, reflecting a more complex and dynamic community structure where interspecies cooperation and competition coexist in a resource-rich environment. Furthermore, all seasonal AMMs co-occurrence networks maintained significant modularity (modularity > 0.4) (Table S5), indicating clear ecological functional compartmentalization. Such modular structures contribute to improved stability, disturbance resistance, and environmental adaptability of the sediment microbial ecosystem.

### Environmental drivers of sedimentary As metabolic genes and AMM composition

**Fig 6:**
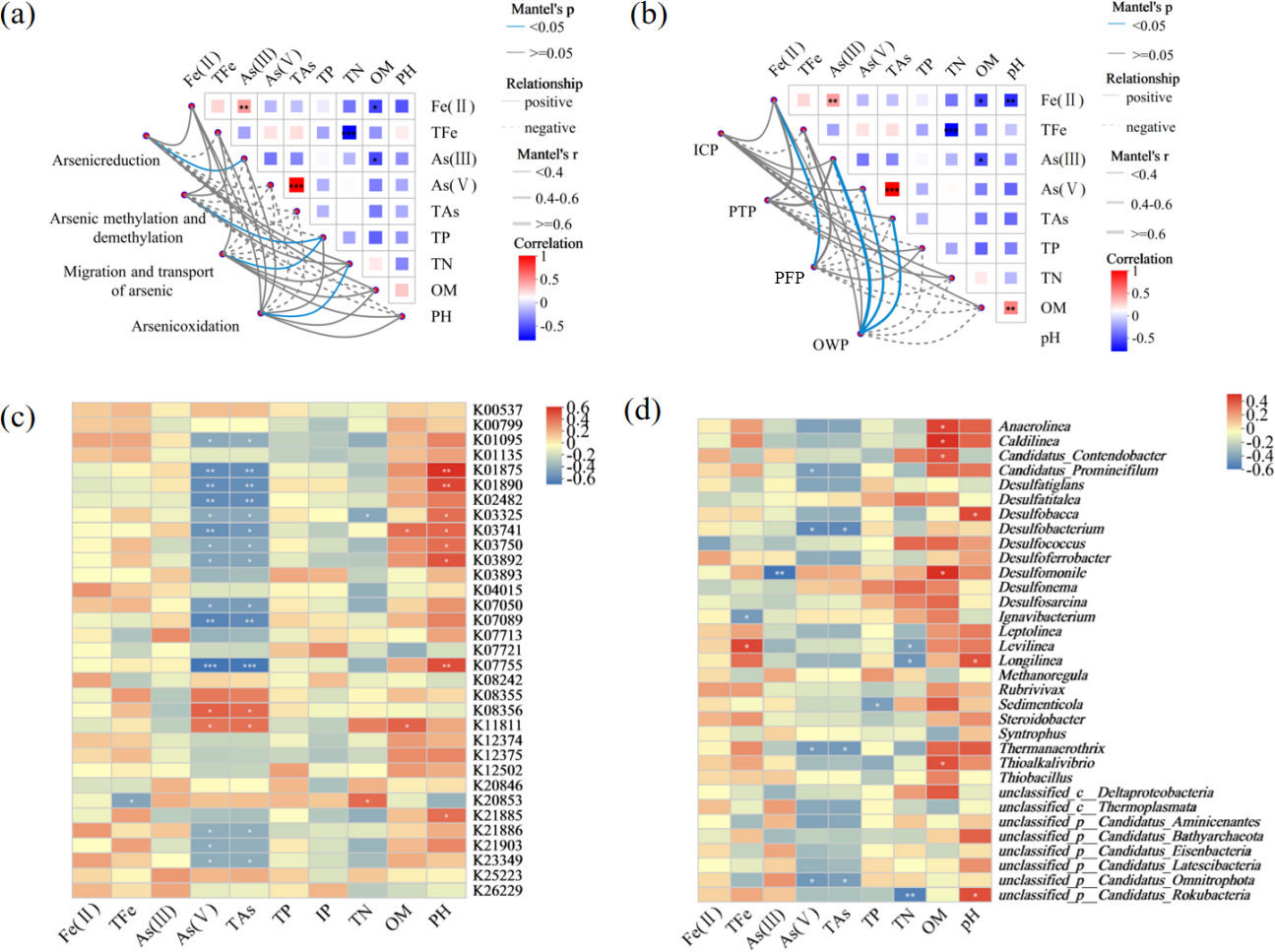
Environmental drivers of sediment As metabolic genes and AMM community composition. Pairwise comparisons between sediment environmental factors and As metabolic genes (**a**) or AMMs community composition (**b**), with a color gradient representing the Spearman correlation coefficient. Partial Mantel tests were used to assess the correlations between As metabolic genes or AMMs community composition and individual environmental factors. Lines in the diagram indicate the correlations between community structure and environmental factors, while the heatmap represents correlations among environmental factors themselves. Edge width corresponds to the Mantel r statistic of distance correlation, and edge color indicates statistical significance based on 999 permutations. Heatmaps showing the correlation between environmental factors and As metabolic genes (**c**) and AMMs taxa (**d**), reflecting the strength and significance of correlations between multiple environmental variables and individual genes or taxa. Asterisks in color blocks indicate significance levels (* 0.01 < *P* ≤ 0.05, ** 0.001 < *P* ≤ 0.01). PFP represents pre-freeze period, ICP represents ice-covered period, PTP represents post-thaw period, and OWP represents open-water period.

Mantel test results indicated that sediment As(III), As(V), TAs, TN, and TP were primary environmental regulators of As metabolic genes (Fig. 6a and c). Specifically, As(III) positively correlated with As reduction genes (*P* < 0.05, r = 0.146), TN positively associated with As oxidation genes (*P* < 0.05, r = 0.165), and TP positively linked to genes involved in As methylation/demethylation and transport (*P* < 0.05, r = 0.133 and 0.180, respectively) (Table S7). For AMM communities, sediment Fe(II), As(III), As(V), TAs, and pH were identified as key environmental drivers during the freeze-thaw process (Fig. 6b and d). Fe(II) was significantly positively correlated with the pre-freezing period community (*P* < 0.05, r = 0.557), whereas As(III), As(V), and TAs showed strong positive correlations with the AMMs community in the open-water period (*P* < 0.05, r = 0.605, 0.586, and 0.573, respectively). In addition, pH exhibited a significant positive correlation with the ice-covered period AMMs community (*P* < 0.05, r = 0.588) (Table S8). These results collectively suggested that As metabolic genes expression is significantly modulated by specific environmental parameters, and different gene types and seasonal AMMs communities are driven by distinct environmental factors. This highlights the dynamic nature of microbial functional responses under the influence of redox shifts, nutrient fluctuations, and As speciation during the seasonal freeze-thaw process.

**Fig 7:**
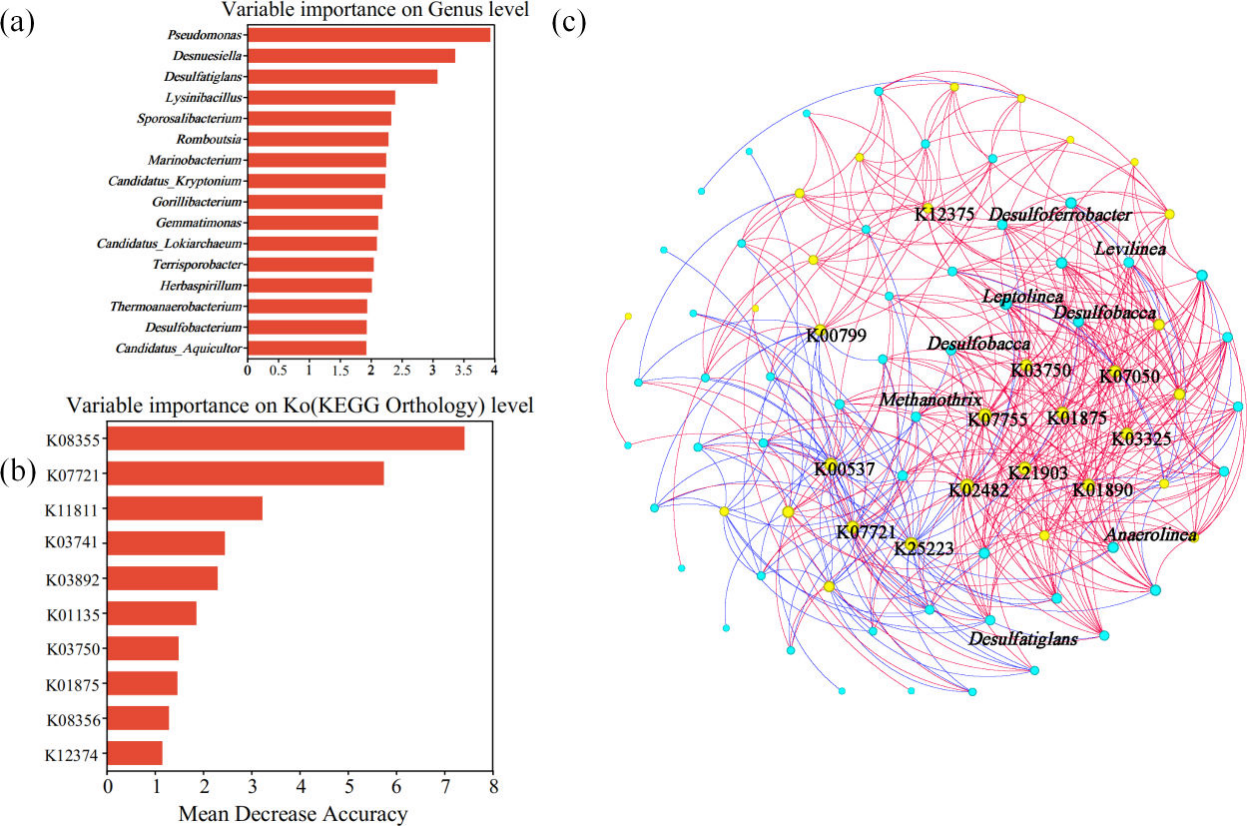
(**a**) Random forest analysis identifying biomarker genera of AMMs in sediments. (**b**) Random forest analysis identifying key functional genes involved in sediment As metabolism. The horizontal axis represents the mean decreased accuracy, with higher values indicating greater importance. (**c**) Co-occurrence network of core genera and functional genes involved in As metabolism in sediments. The basic topological characteristics of the network are summarized in Table S6.

Random Forest modeling identified key predictors significantly influencing sedimentary As-metabolizing microorganisms and functional genes (Fig. 7). The genus *Pseudomonas* demonstrated the highest feature importance—its members, widely distributed in sediments and frequently harboring functional genes (*aioA* and *arsC*), represent quintessential As-oxidizing/resistant microorganisms. In addition, anaerobic genera such as *Desulfatiglans* and *Desnuesiella* ranked among the top predictors. These taxa may contribute to the transformation of As(V) to As(III) by providing reductive substrates or facilitating cooperative metabolism through sulfate reduction pathways. Multiple KEGG functional genes (*aioB*, *arsR*, and *arsH*) exhibited high predictive importance, corresponding to key enzymes involved in As redox cycling, indicating microbial engagement in As redox cycling via encoded biotransformation pathways.

Variable importance analysis based on the Random Forest model revealed that physicochemical parameters, functional genes, and core microbial taxa are key predictors governing the structure and function of AMMs in sediments (Fig. S3). OM and pH emerged as the most important predictors in the model, indicating that they are critical drivers of both metabolic gene expression and their host microbial communities (37). OM acts as a carbon substrate directly affecting microbial metabolic activity, while pH indirectly influences microbial structure and function by modulating redox conditions and the speciation of As (As(III)/As(V)). This aligns with recent findings demonstrating significant associations between As metabolic genes and environmental factors such as As, N, P, and pH (38). In addition to key taxa and genes, Fe(II) was also identified as a strong predictor of sediment As metabolism-related genes (Fig. S3). However, its importance was relatively lower than expected, suggesting that microbial responses to As metabolism may be more constrained by organic matter availability and community composition rather than directly driven by iron redox processes. Nevertheless, it should be noted that the speciation and concentration of iron could still exert indirect effects on AMM activity by influencing As binding forms, solubility, and interface reactivity.

## DISCUSSION

### Impact of the freeze-thaw process on As metabolism genes in sediments

The abundances of As oxidation genes *aioB* in the sediments of WLSH decreased significantly during the ice-covered period (*P* < 0.05) (Fig. 2). This decline may be attributed to a series of ecological changes typically occurring during the ice-covered period, including markedly reduced temperatures, restricted oxygen exchange between the water and atmosphere, and diminished OM input. These environmental constraints could suppress the growth and metabolic activity of aerobic As-oxidizing microorganisms, thereby leading to a lower abundance of the *aioB* genes they harbor. As typical key functional genes involved in As oxidation, reduced relative abundance of *aioB* may indicate weakening of the microbially driven As(III)-to-As(V) oxidation pathway, potentially leading to the accumulation of As(III) or depressed oxidation rates under ice-covered conditions.

Reduction/resistance functions were maintained year-round; however, the significant period effect observed for *arsR* (*P* < 0.05) suggests that As stress-response regulation is seasonally modulated, with the strongest signal occurring in the open-water period. Within the migration/transport module, *arsB* exhibited a significant period effect (*P* < 0.05), with higher abundances observed during both the ice-covered and open-water periods compared to the post-thaw phase. This pattern implies that efflux/transport potential is selectively adjusted across seasonal stages, likely reflecting shifts in As exposure and redox constraints associated with ice cover and subsequent transitions, which may demand distinct detoxification strategies at different times of the year. In contrast, the methylation/demethylation module remained relatively stable. *ArsM* remained consistently abundant across all periods, whereas *arsI* persisted at low levels with minimal fluctuation, indicating a persistent background potential for methylation throughout the freeze-thaw cycle. Collectively, these findings point to a freeze-thaw-driven functional reorganization of the sediment’s As metabolome. This reorganization is characterized not by uniform shifts across all pathways, but by a targeted reallocation of genetic investment: seasonal enhancement of oxidation potential, stage-specific tuning of regulatory and transport functions (*arsR, arsB*), and the maintenance of a stable methylation backdrop (*arsM*). Such a strategy likely reflects adaptive microbial responses to the cyclic redox, temperature, and solute fluctuations that define seasonal ice dynamics in cold-region lakes.

### Impacts of the freeze-thaw process on AMMs

The seasonal succession of microbial community composition highlights dynamic interactions between abiotic conditions and As-cycling taxa. During the ice-covered period, relative decreases in the abundances of key phyla such as Pseudomonadota, Bacteroidota, and Chloroflexota were observed compared to other seasons. Given that many taxa within these groups contribute to As oxidation and reduction pathways, their reduced representation may reflect a potential limitation of As-related microbial metabolism under persistently cold, dark, and often anoxic conditions, even if the observed trends did not reach statistical significance. This interpretation is further supported by functional network and random forest analyses, which identified Pseudomonadota, Bacteroidota, and Actinomycetota as key taxa in sediment As cycling (Fig. 7). When combined with the NMDS analysis of community structure (Fig. S1a), these results indicate that freeze-thaw transitions drive significant compositional restructuring within sediment microbial assemblages. Notably, the α-diversity indices of AMMs remained statistically stable across seasonal transitions (Fig. S2), suggesting a degree of functional redundancy or resilience within the As-cycling community. Such stability may enable the maintenance of core metabolic functions, such as oxidation, reduction, and transport, even as the relative abundances of specific taxa fluctuate in response to environmental change.

At the genus level, As-functional microbial community transitions aligned closely with seasonal redox dynamics. During the pre-freeze period, more stable oxic to suboxic conditions corresponded with a greater relative abundance of known As-oxidizing bacteria and facultative anaerobes such as Thiobacillus, potentially favoring the stabilization of As in the less mobile As(V) form. In contrast, the ice-covered period was characterized by increasingly reducing conditions and elevated relative abundances of methanogenic taxa (e.g., Methanothrix) and sulfate-reducing bacteria (e.g., Desulfobacca and Desulfobacterium) (Fig. 3e). Although not always statistically enriched, these functional guilds reflect an environment conducive to reductive processes. In particular, the oxidation of organic matter under these reducing conditions can drive the microbial reduction of As(V) to the more mobile and toxic As(III), a process that may occur concomitantly with iron and sulfate reduction.

During the post-thaw period, ice melting releases organic matter and nutrients into surface sediments, coinciding with an increase in the representation of heterotrophic genera such as Anaerolinea. These taxa may enhance the availability of substrates that support syntrophic interactions and metabolic coupling, thereby stimulating As transformation pathways. In the open-water period, microbial communities tend to shift toward assemblages dominated by aerobic and facultative taxa, with As-oxidizing bacteria such as Thiobacillus again becoming more abundant, suggesting a renewed dominance of oxidative processes that favor the stabilization of arsenic as As(V).

Collectively, these seasonal patterns highlight how freeze-thaw cycles restructure microbial assemblages, steer As speciation pathways, and act as critical ecological regulators of biogeochemical cycling in lacustrine sediments.

### Interrelationships among sediment As metabolic genes, AMMs, and environmental factors

Mantel analysis results (Fig. 6) showed significant positive correlations between As metabolic gene abundances and nutrients (TN and TP) in WLSH sediments, indicating pivotal roles of nutrient availability in regulating microbially-driven As cycling. The significant positive correlation between As oxidation genes and TN may indicate that sufficient nitrogen availability likely enhances microbial metabolism and growth, potentiating As oxidation capacity. In contrast, As reduction genes were significantly positively correlated with As(III) concentrations, possibly reflecting an induction effect of As(III) on gene expression to facilitate microbial detoxification or energy acquisition. Additionally, TP showed significant positive correlations with As methylation/demethylation genes and transport-related genes. On one hand, increased TP may influence microbial metabolic pathways and regulate the expression of arsenic functional genes; on the other hand, phosphorus indirect mediation via competitive adsorption that alters As bioavailability (39, 40).

During the pre-freeze period, AMMs showed a significant positive correlation with Fe(II) (Fig. 6), indicating that critical ecological roles of iron reduction during this stage. At the onset of ice formation, reduced water body disturbance and the upward migration of the redox interface create a relatively stable anaerobic environment favorable for Fe-reducing bacteria that use Fe(III) as an electron acceptor, thereby enhancing metabolic cooperation and interactions within the microbial community. Previous studies have shown that indigenous Fe-reducing bacteria can promote reductive dissolution of Fe hydroxides (8, 18, 41, 42), which facilitates the release and mobilization of adsorbed As. In the ice-covered period, pH was the only environmental factor significantly correlated with the AMMs community structure. Under low temperature and reduced disturbance conditions, microbial metabolic activities are suppressed, making community structure more sensitive to subtle environmental changes. Elevated pH may alter the availability of dissolved organic matter and nutrients, thereby restructuring ecological interactions. During this period, sediments are strongly reducing, with increased relative abundance of anaerobic As-reducing bacteria and Fe/S-reducing bacteria such as *Anaerolinea*, *Desulfobacca*, and *Desulfobacterium*. These microbes reduce metal oxides and As(V), promoting the accumulation of the more mobile and toxic As(III), thus increasing As mobility and toxicity. In the open-water period, As(III), As(V), and TAs showed significant positive correlations with the AMMs structure (*P* < 0.05), indicating that As concentration and speciation critically regulate microbial community assembly during this phase. The warming water temperature, increased disturbance, and sufficient dissolved oxygen create favorable conditions for the coexistence and metabolic cooperation of various As-metabolizing bacteria, including both oxidizers and reducers. The aerobic environment during the open-water period promoted the dominance of As-oxidizing genera such as *Thiobacillus* and *Thioalkalivibrio*, which catalyze the conversion of As(III) into As(V), thereby favoring As immobilization and reducing its mobility and bioavailability in the water column.

Spearman correlation analysis further corroborated strong freeze-thaw-driven restructuring of As-metabolizing microbial communities, revealing significant associations between multiple genera and As/environmental factors. Notably, typical sulfate-reducing bacteria such as *Desulfobacca*, *Desulfobacterium*, and *Desulfatiglans* were enriched under reducing conditions. These genera not only modulate sediment redox states via sulfate reduction but also harbor key As reduction genes such as *arsC* or *arrA*, thereby enhancing As mobility and ecological risk (41, 43). In contrast, sulfur-oxidizing bacteria like *Thiobacillus* and *Thioalkalivibrio* proliferating in oxidizing environments, potentially facilitating the oxidation of As(III) to As(V) under aerobic conditions via redox-coupled mechanisms, thus contributing to As stabilization and detoxification. Additionally, the abundance of Syntrophus and Methanoregula, both associated with OM, suggesting that OM input may indirectly enhance the activity of As-metabolizing microorganisms by serving as electron donors or intermediates in microbial metabolism.

### Conclusions

This study systematically elucidated the spatiotemporal response mechanisms of AMMs and their functional genes to seasonal freeze-thaw processes in WLSH sediments. The depression of *aioB* during the ice-covered period and the increase in *arxR/arxS* toward the open-water period are consistent with reduced representation of oxidation potential under ice cover and greater representation under ice-free conditions. Collectively promoting As(V)-to-As(III) transformation and elevating As mobility and ecological risks. Concurrently, AMMs exhibited distinct stage-dependent succession, with anaerobic As-reducing bacteria dominating in the ice-covered period and As-oxidizing bacteria prevailing in the open-water period, reflecting high sensitivity to redox fluctuations. Despite compositional shifts, the α-diversity of AMMs remained relatively stable across the freeze-thaw process, suggesting that these communities possess high functional redundancy and ecological resilience under environmental stress. Random forest and Mantel analyses further revealed that As(III), As(V), TAs, TP, TN, pH, and Fe(II) significantly regulated the expression of functional As genes and the assembly of AMM communities by modulation of electron donor/acceptor availability and elemental coupling mechanisms. Enhanced OM inputs during post-thaw and open-water stages stimulated heterotrophic activity and microbial interactions, intensifying As biotransformation.

In summary, seasonal freeze-thaw processes profoundly influence As transformation pathways and environmental fate in lake sediments by regulating microbial metabolic functions, altering redox regimes, and restructuring community interaction networks. Meanwhile, environmental factors such as pH, TN, and TP synergistically shape AMMs’ structure and function through metabolic co-regulation. This work provides a theoretical foundation for understanding lake As pollution dynamics under freeze-thaw conditions and scientific support for ecological remediation and risk mitigation strategies.

## SUPPLEMENTAL MATERIAL

File S1 aem.00485-26-s0001.xlsx The sample type used in this study was lake sediment.

Supplemental tables and figures aem.00485-26-s0002.docx Tables S1 to S8, and Fig S1 to S3.

ASM does not own the copyrights to Supplemental Material that may be linked to, or accessed through, an article. The authors have granted ASM a non-exclusive, world-wide license to publish the Supplemental Material files. Please contact the corresponding author directly for reuse.
