# Supplementary material for: Seasonal freeze-thaw drives arsenic metabolism dynamics in sediments of a cold-region lake: microbial gene and community responses
Source: Appl Environ Microbiol. 2026 May 5;92(6):e00485-26. doi: 10.1128/aem.00485-26 (PMC13274347; doi:10.1128/aem.00485-26)
Supplement: Supplemental tables and figures — Tables S1 to S8, and Fig S1 to S3. [file aem.00485-26-s0002.docx]

**Supplementary Materials**

**Seasonal Freeze-Thaw Drives Arsenic Metabolism Dynamics in Sediments of a Cold-Region Lake: Microbial Gene and Community Responses**

**Wenjing Shi^a,b*^[[1]](#footnote-1)^^, Haoran Xu^a,b^, Weiping Li ^a,b^, Jin Xu^a,b^, Yan Qin^a,b^, Long Bai^a,b^**

a School of Energy and Environment, Inner Mongolia University of Science & Technology, BaoTou 014010, China

b Collaborative Innovation Center for Ecological Protection and Comprehensive Utilization of the

Inner Mongolia Section of the Yellow River Basin, Inner Mongolia University of Science & Technology, BaoTou 014010, China

**S1 Community composition of sediment AMMs**

At the phylum level, the dominant archaeal phyla were Euryarchaeota, Candidatus_Thermoplasmatota, Candidatus_Bathyarchaeota, Nanoarchaeota, and Candidatus_Thorarchaeota, which together accounted for 86.24–87.06% of all archaeal sequences. The top five bacterial phyla were Pseudomonadota, Thermodesulfobacteriota, Chloroflexota, Bacteroidota, and Candidatus_Aminicenantes, comprising 67.23–69.95% of all bacterial sequences. For fungi, only four phyla were detected: Ascomycota, Chytridiomycota, Basidiomycota, and Mucoromycota. Across the seasonal freeze–thaw process, Pseudomonadota consistently dominated the As-metabolizing microbial (AMM) community in the sediments. Similar dominance of this phylum in sedimentary environments has also been reported in comparable ecosystems (1). Notably, this phylum includes several well-known As-metabolizing genera, such as Pseudomonas and Shewanella, which are considered to play key roles in both As(III) oxidation and As(V) reduction.

At the genus level, the five most abundant archaeal genera were *unclassified_p_Candidatus_Bathyarchaeota*, *unclassified_c_Thermoplasmata,Methanoregula*, *unclassified_p__Candidatus_Thermoplasmatota,* and *unclassified_p_Euryarchaeota*. For bacteria, the dominant genera included *Anaerolinea*, *Thiobacillus*, *unclassified_p__Candidatus_Aminicenantes*, *unclassified_c__Deltaproteobacteria*, and *Desulfosarcina*.

**Table S1** Post-quality control sequence statistics

| Samples ID | Clean reads | Clean base(bp) | Percent in raw reads(%) | Percent in raw bases(%) |
| --- | --- | --- | --- | --- |
| QW1 | 89364082 | 13437668438 | 98.55 | 98.14 |
| QW2 | 87489322 | 13156908720 | 98.76 | 98.36 |
| QW3 | 90194464 | 13570146566 | 98.69 | 98.33 |
| QW4 | 83803420 | 12601561223 | 98.61 | 98.20 |
| QW5 | 87202398 | 13115396941 | 98.90 | 98.51 |
| QW6 | 86290458 | 12977754472 | 98.89 | 98.50 |
| QW7 | 85501938 | 12860374357 | 98.58 | 98.19 |
| DW1 | 108519470 | 16337565507 | 98.32 | 98.03 |
| DW2 | 91636448 | 13805337587 | 97.59 | 97.37 |
| DW3 | 90082208 | 13566211684 | 97.71 | 97.45 |
| DW4 | 100967614 | 15205861047 | 97.83 | 97.57 |
| DW5 | 100067266 | 15072069812 | 97.96 | 97.72 |
| DW6 | 88304816 | 13299552377 | 97.68 | 97.42 |
| DW7 | 92470280 | 13922831125 | 98.00 | 97.72 |
| CW1 | 93942784 | 14151682079 | 98.24 | 98.01 |
| CW2 | 85644630 | 12905420836 | 98.33 | 98.12 |
| CW3 | 93476328 | 14084604389 | 98.16 | 97.95 |
| CW4 | 87648092 | 13205167585 | 98.00 | 97.78 |
| CW5 | 83739606 | 12617137851 | 98.44 | 98.23 |
| CW6 | 84969384 | 12801258024 | 98.45 | 98.22 |
| CW7 | 85313302 | 12853456827 | 97.94 | 97.72 |
| XW1 | 84817908 | 12784041969 | 98.92 | 98.74 |
| XW2 | 84180460 | 12689312081 | 99.09 | 98.92 |
| XW3 | 87328242 | 13159323752 | 98.94 | 98.73 |
| XW4 | 88997424 | 13413901267 | 98.94 | 98.75 |
| XW5 | 90854350 | 13692235709 | 98.89 | 98.70 |
| XW6 | 95307262 | 14365535760 | 99.02 | 98.84 |
| XW7 | 84687514 | 12763920925 | 98.98 | 98.79 |

**Table S2** The KO number, function descriptions, gene name and classification of the investigated genes related to sediment As cycle.

| Classification | KO number | Details for gene function | Corresponding gene |
| --- | --- | --- | --- |
| Arsenic oxidation | K09452 | unknown | *arxB*(2) |
|  | K02482 | a component of the two-component signal transduction system | *arxS* (3) |
|  | K03750 | biosynthesis of the molybdenum cofactor for AioAB | *moeA* (3) |
|  | K07713、K07714 | regulation of the expression of the aio/arx operon | *arxR* (4) |
|  | K08355 | oxidation of As(III) | *aioB* (3) |
|  | K08356 | oxidation of As(III) | *aioA* (3) |
|  | K11811 | oxidation of trimethylated and aromatic arsenic compounds, and reduction of Cr and Fe | *arsH* (5) |
|  | K23349 | involved in As(III) oxidation | *arxA* (4) |
| Arsenic reduction | K00537、K03741、K18701 | reduction of As(V) | *arsC* (6) |
|  | K00799 | regeneration of reduced glutathione (GSH) | *GstB* (7) |
|  | K03892、K07721、K21885、K21886、K21903 | regulation of the expression of the arr/ars operons | *arsR* (8) |
|  | K03069、K20846、K20850、K20853 | reduction of As(V) | *arrA* (9) |
|  | K04439、K28467 | reduction of As(V) | *arrB*(10) |
| Arsenic methylation and demethylation | K01875、K08242、K12502、K07755 | Methylated forms of arsenic | *arsM* (11) |
|  | K12375 | Catalyze the demethylation of trimethylated arsenic compounds | *arsI* (12) |
| Migration and transport of arsenic | K01095 | Recognition and transport of thiol-metal complexes | *pgpA* (13) |
|  | K01135 | Efflux of As(III) out of the cell | *arsB* (14) |
|  | K01890、K03893 | Transport of As(III) | *arsA* (15) |
|  | K03325 | Efflux of As(III) out of the cell | *ACR3* (16) |
|  | K07050、K12374 | Transfer of trivalent metalloids to ArsA | *arsD* (15) |
|  | K07089 | Efflux of trimethylarsine (TMA) from the cell | *arsP* (17) |
|  | K25223 | Efflux of organic arsenic from the cell | *arsJ* (18) |

**Table S3** The sediments biogeochemical properties during seasonal freeze–thaw process. Different letters in the same row indicate significant differences among the different stages of the freeze–thaw process at the P < 0.05 level. (Duncan’s test). Note: PFP represent pre-freeze period, ICP represent ice-covered period, PTP represent post-thaw period, OWP represent open-water period.

| Sediment properties | PFP | ICP | PTP | OWP |
| --- | --- | --- | --- | --- |
| As(Ⅲ) (mg kg^−1^) | 0.312±0.11b | 0.448±0.91a | 0.407±0.13ab | 0.299±0.07b |
| As(Ⅴ) (mg kg^−1^) | 14.17±2.18a | 12.27±1.56ab | 13.33±1.94ab | 11.48±2.91b |
| TAs (mg kg−^1^) | 14.48±2.18a | 12.72±1.55ab | 13.73±1.88ab | 11.78±2.9b |
| Fe(Ⅱ) (g kg^−1^) | 3.52±0.94ab | 4.3±1.34a | 3.86±0.89a | 2.7±0.20b |
| TFe (g kg^−1^) | 29.93±3.77a | 27.66±3.12a | 29.25±2.04a | 28.46±3.56a |
| pH | 7.02±0.03b | 6.97±0.08b | 7.06±0.15ab | 7.16±0.06a |
| TN (g kg^−1^) | 3.37±1.44a | 3.6±2.16a | 3.77±0.74a | 3.61±0.90a |
| TP(mg kg^−1^) | 553.95±81.03a | 530.64±50.21a | 410.64±65.36b | 430.47±40.67b |
| OM(g kg^−1^) | 44.96±5.43b | 28.31±9.50c | 45.83±5.57b | 74.93±11.25a |

Note: Arsenite (As(Ⅲ)), Arsenate (As(Ⅴ)), Total Arsenic (TAs), Total Nitrogen (TN), Total Phosphorus (TP), Organic Matter (OM).

| Topological properties | Explanation | PFP | ICP | PTP | OWP |
| --- | --- | --- | --- | --- | --- |
| Average degree | The average number of edges connected to nodes in a network | 4.333 | 4.375 | 4.154 | 3.677 |
| Graph density | The ratio of the number of edges and the maximal number of edges in a network | 0.188 | 0.141 | 0.166 | 0.123 |
| Modularity | The strength of the division of a network into functionally independent modules | 0.329 | 0.474 | 0.592 | 0.483 |
| Nodes | The number of nodes. | 24 | 32 | 26 | 31 |
| Edges | The number of edges or connections. | 52 | 70 | 54 | 57 |
| Positive | The negative relationships | 61.54% | 68.57% | 74.07% | 82.46% |
| Negative | The positive relationships | 38.46% | 31.43% | 25.93% | 17.54% |

**Table S4** Basic topological properties of gene occurrence networks with different phases of the freeze-thaw process. Note: PFP represent pre-freeze period, ICP represent ice-covered period, PTP represent post-thaw period, OWP represent open-water period.

**Table S5** Basic topological properties of AMMs occurrence networks with different period of the freeze-thaw process. Note: PFP represent pre-freeze period, ICP represent ice-covered period, PTP represent post-thaw period, OWP represent open-water period.

| Topological properties | Explanation | PFP | ICP | PTP | OWP |
| --- | --- | --- | --- | --- | --- |
| Average degree | The average number of edges connected to nodes in a network | 11.616 | 18.245 | 8.633 | 17.711 |
| Graph density | The ratio of the number of edges and the maximal number of edges in a network | 0.119 | 0.188 | 0.089 | 0.184 |
| Modularity | The strength of the division of a network into functionally independent modules | 0.463 | 0.435 | 0.543 | 0.376 |
| Nodes | The number of nodes. | 99 | 98 | 98 | 97 |
| Edges | The number of edges or connections. | 575 | 894 | 423 | 859 |
| Positive | The negative relationships | 65.22% | 58.05% | 63.83% | 50.64% |
| Negative | The positive relationships | 34.78% | 41.95% | 36.17% | 49.36% |

**Table S6** Basic topological properties of functional occurrence network with different period of the freeze-thaw process.

| Topological properties | Explanation | Value |
| --- | --- | --- |
| Average degree | The average number of edges connected to nodes in a network | 11.185 |
| Graph density | The ratio of the number of edges and the maximal number of edges in a network | 0.14 |
| Modularity | The strength of the division of a network into functionally independent modules | 0.345 |
| Nodes | The number of nodes | 81 |
| Edges | The number of edges or connections | 453 |

Table S7 The Mantel test between the classification of sediments As metabolic genes and sediment properties. Fe(Ⅱ): Ferrous iron; TFe: total iron; As(Ⅲ): trivalent arsenic; As(Ⅴ): pentavalent arsenic; TAs: total arsenic; TP: total phosphorus; TN: total nitrogen; OM: organic matter.

| Classification | soil properties | r | p.value |
| --- | --- | --- | --- |
| Genes involved in  As oxidation | Fe(Ⅱ) | 0.018 | 0.346 |
|  | TFe | 0.033 | 0.364 |
|  | As(Ⅲ) | 0.022 | 0.362 |
|  | As(Ⅴ) | -0.086 | 0.823 |
|  | TAs | -0.085 | 0.815 |
|  | TP | 0.061 | 0.203 |
|  | TN | 0.165 | 0.049 |
|  | OM | 0.007 | 0.459 |
|  | pH | 0.073 | 0.231 |
| Genes involved in  As reduction | Fe(Ⅱ) | 0.048 | 0.215 |
|  | TFe | -0.046 | 0.699 |
|  | As(Ⅲ) | 0.146 | 0.031 |
|  | As(Ⅴ) | -0.006 | 0.484 |
|  | TAs | -0.005 | 0.489 |
|  | TP | -0.004 | 0.467 |
|  | TN | 0.018 | 0.409 |
|  | OM | 0.125 | 0.084 |
|  | pH | -0.014 | 0.527 |
| Genes involved in  As methylation and demethylation | Fe(Ⅱ) | 0.060 | 0.216 |
|  | TFe | 0.011 | 0.424 |
|  | As(Ⅲ) | -0.007 | 0.485 |
|  | As(Ⅴ) | -0.070 | 0.741 |
|  | TAs | -0.068 | 0.731 |
|  | TP | 0.180 | 0.014 |
|  | TN | 0.000 | 0.450 |
|  | OM | -0.040 | 0.654 |
|  | pH | -0.118 | 0.870 |
| Genes involved in  Migration and transport of As | Fe(Ⅱ) | 0.060 | 0.186 |
|  | TFe | -0.043 | 0.670 |
|  | As(Ⅲ) | -0.006 | 0.507 |
|  | As(Ⅴ) | -0.015 | 0.529 |
|  | TAs | -0.006 | 0.488 |
|  | TP | 0.133 | 0.033 |
|  | TN | 0.011 | 0.446 |
|  | OM | 0.058 | 0.269 |
|  | pH | 0.085 | 0.184 |

Table S8 The Mantel test between the AMMs and sediment properties.Fe(Ⅱ): Ferrous iron; TFe: total iron; As(Ⅲ): trivalent arsenic; As(Ⅴ): pentavalent arsenic; TAs: total arsenic; TP: total phosphorus; TN: total nitrogen; OM: organic matter.

| period | soil properties | r | p.value |
| --- | --- | --- | --- |
| PFP | Fe(Ⅱ) | 0.557 | 0.040 |
|  | TFe | 0.501 | 0.098 |
|  | As(Ⅲ) | -0.179 | 0.832 |
|  | As(Ⅴ) | -0.243 | 0.739 |
|  | TAs | -0.253 | 0.760 |
|  | TP | 0.264 | 0.195 |
|  | TN | -0.303 | 0.874 |
|  | OM | -0.099 | 0.658 |
|  | pH | 0.030 | 0.384 |
| ICP | Fe(Ⅱ) | 0.179 | 0.155 |
|  | TFe | -0.116 | 0.673 |
|  | As(Ⅲ) | 0.222 | 0.166 |
|  | As(Ⅴ) | 0.299 | 0.099 |
|  | TAs | 0.273 | 0.122 |
|  | TP | -0.301 | 0.875 |
|  | TN | 0.109 | 0.324 |
|  | OM | 0.127 | 0.269 |
|  | pH | 0.588 | 0.013 |
| PTP | Fe(Ⅱ) | 0.504 | 0.084 |
|  | TFe | -0.279 | 0.816 |
|  | As(Ⅲ) | 0.422 | 0.062 |
|  | As(Ⅴ) | -0.203 | 0.804 |
|  | TAs | -0.286 | 0.884 |
|  | TP | 0.108 | 0.282 |
|  | TN | 0.248 | 0.164 |
|  | OM | -0.044 | 0.602 |
|  | pH | 0.083 | 0.402 |
| OWP | Fe(Ⅱ) | 0.227 | 0.237 |
|  | TFe | 0.044 | 0.391 |
|  | As(Ⅲ) | 0.605 | 0.027 |
|  | As(Ⅴ) | 0.586 | 0.024 |
|  | TAs | 0.573 | 0.024 |
|  | TP | -0.065 | 0.564 |
|  | TN | -0.100 | 0.583 |
|  | OM | -0.277 | 0.777 |
|  | pH | -0.204 | 0.819 |


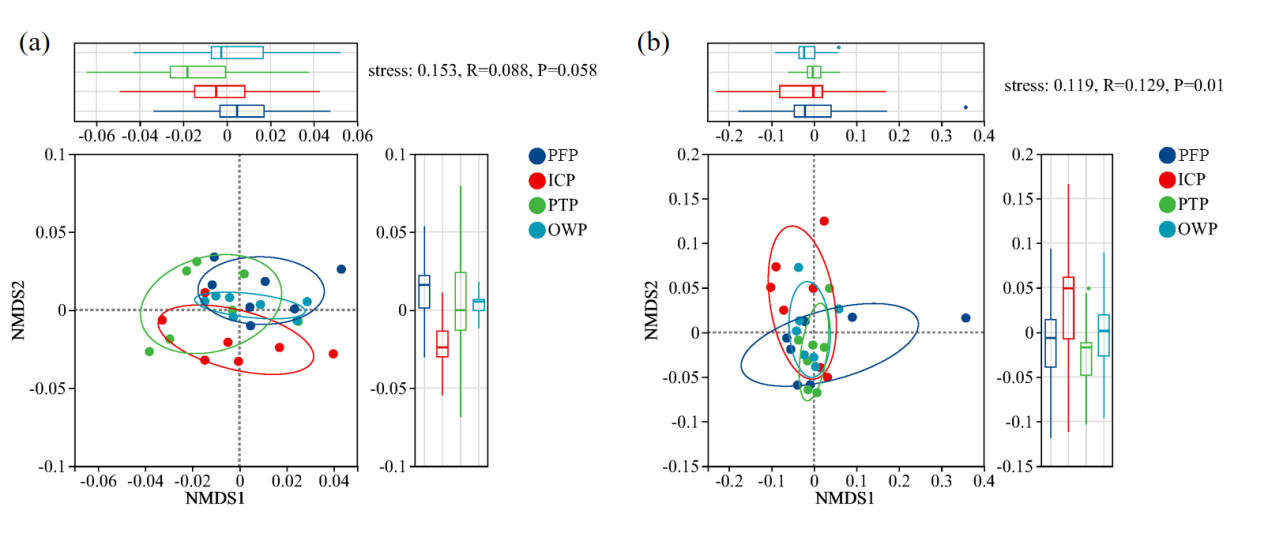
**Fig. S1** Bray-Curtis based NMDS analysis of (a) sediments As metabolic genes and (b) AMMs in different period of the freeze-thaw process. Note: PFP represent pre-freeze period, ICP represent ice-covered period, PTP represent post-thaw period, OWP represent open-water period.


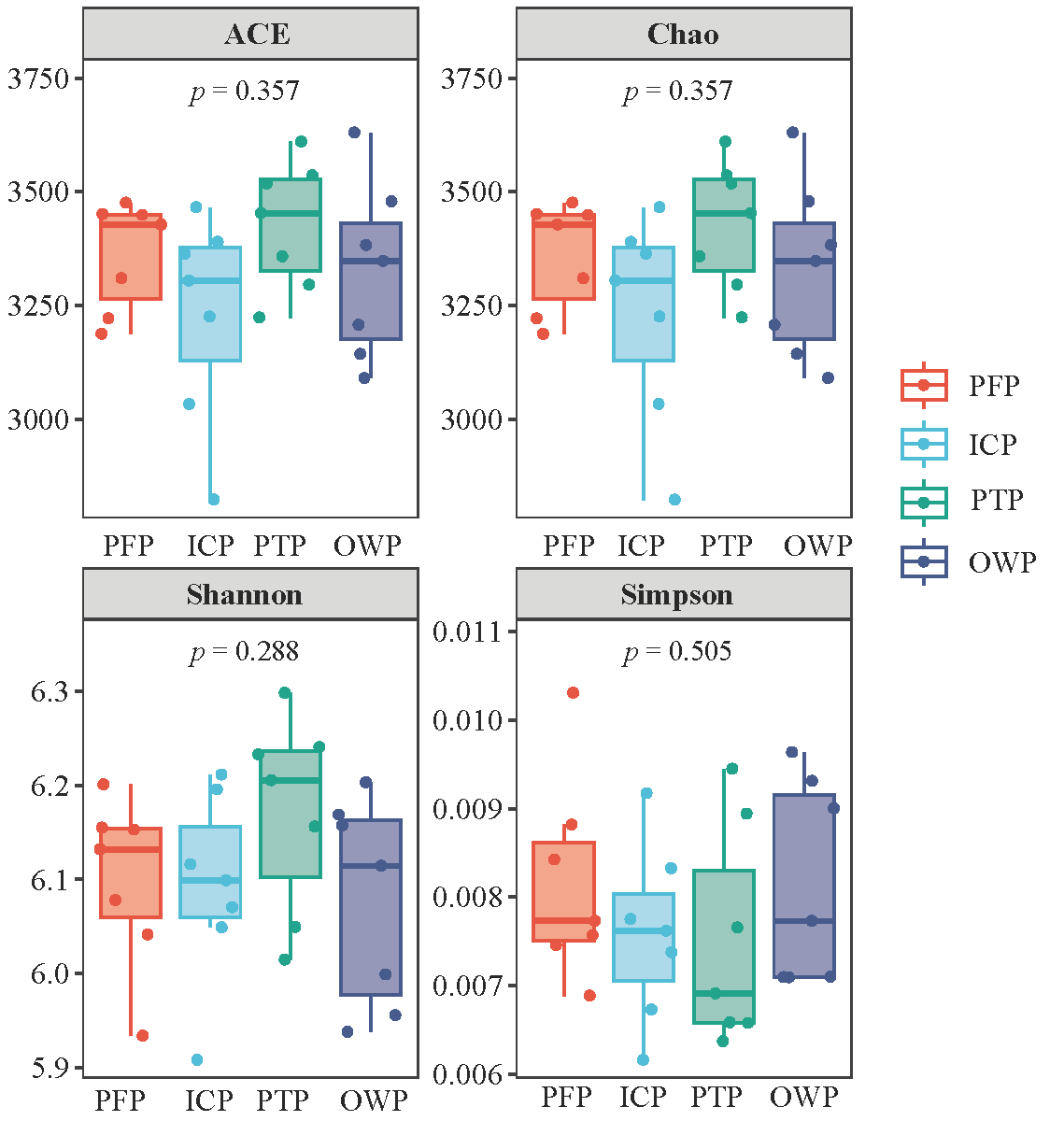


**Fig.S2** Alpha diversity indices of As-related functional microbial communities.((a) ACE index; (b) Chao index; (c) Shannon index; (d) Simpson index). Note: PFP represent pre-freeze period, ICP represent ice-covered period, PTP represent post-thaw period, OWP represent open-water period.


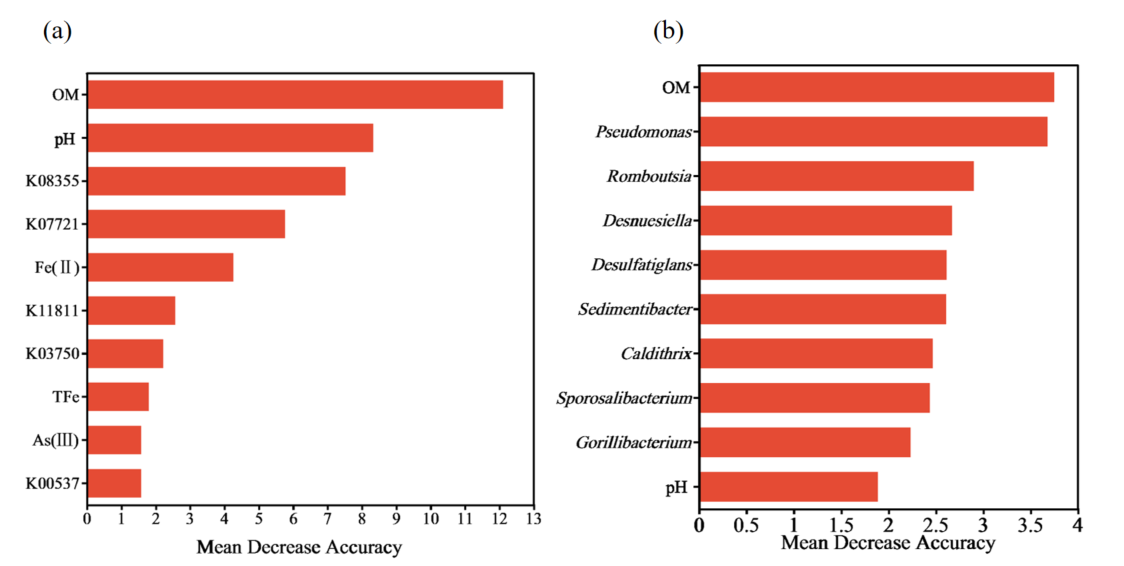


**Fig.S3** Importance ranking of (a) genes and environmental factors, and (b) taxa and environmental factors. The horizontal axis represents the Mean Decrease Accuracy, with higher values indicating greater importance. The vertical axis shows the names of genes or taxa, ranked by their importance.

**References**：

1.Wang, Yanhong, Li, P., Li, B., Webster, G., Weightman, A.J., Jiang, Z., Jiang, D., Deng, Y., Wang, Yanxin, 2014. Bacterial Diversity and Community Structure in High Arsenic Aquifers in Hetao Plain of Inner Mongolia, China. Geomicrobiology Journal 31, 338–349.

2.Liu, G., Liu, M., Kim, E.-H., Maaty, W.S., Bothner, B., Lei, B., Rensing, C., Wang, G., McDermott, T.R., 2012. A periplasmic arsenite-binding protein involved in regulating arsenite oxidation. Environ Microbiol 14, 1624–1634.

3.Sardiwal, S., Santini, J.M., Osborne, T.H., Djordjevic, S., 2010. Characterization of a two-component signal transduction system that controls arsenite oxidation in the chemolithoautotroph NT-26. FEMS Microbiol Lett 313, 20–28.

4.Zargar, K., Conrad, A., Bernick, D.L., Lowe, T.M., Stolc, V., Hoeft, S., Oremland, R.S., Stolz, J., Saltikov, C.W., 2012. ArxA, a new clade of arsenite oxidase within the DMSO reductase family of molybdenum oxidoreductases. Environmental Microbiology 14, 1635–1645.

5.Chen, J., Bhattacharjee, H., Rosen, B.P., 2015. ArsH is an organoarsenical oxidase that confers resistance to trivalent forms of the herbicide monosodium methylarsenate and the poultry growth promoter roxarsone. Mol Microbiol 96, 1042–1052.

6.Mukhopadhyay, R., Rosen, B.P., 2002. Arsenate reductases in prokaryotes and eukaryotes. Environ Health Perspect 110 Suppl 5, 745–748.

7.Chrysostomou, C., Quandt, E.M., Marshall, N.M., Stone, E., Georgiou, G., 2015. An alternate pathway of arsenate resistance in E. coli mediated by the glutathione S-transferase GstB. ACS Chem Biol 10, 875–882.

8.Van Lis, R., Nitschke, W., Duval, S., Schoepp-Cothenet, B., 2013. Arsenics as bioenergetic substrates. Biochim Biophys Acta 1827, 176–188.

9.Afkar, E., Lisak, J., Saltikov, C., Basu, P., Oremland, R.S., Stolz, J.F., 2003. The respiratory arsenate reductase from Bacillus selenitireducens strain MLS10. FEMS Microbiol Lett 226, 107–112.

10.Krafft, T., Macy, J.M., 1998. Purification and characterization of the respiratory arsenate reductase of Chrysiogenes arsenatis. Eur J Biochem 255, 647–653.

11.Qin, J., Rosen, B.P., Zhang, Y., Wang, G., Franke, S., Rensing, C., 2006. Arsenic detoxification and evolution of trimethylarsine gas by a microbial arsenite S-adenosylmethionine methyltransferase. Proc Natl Acad Sci U S A 103, 2075–2080.

12.Yoshinaga, M., Rosen, B.P., 2014. A C⋅As lyase for degradation of environmental organoarsenical herbicides and animal husbandry growth promoters. Proc Natl Acad Sci U S A 111, 7701–7706.

13.Grondin, K., Haimeur, A., Mukhopadhyay, R., Rosen, B.P., Ouellette, M., 1997. Co-amplification of the gamma-glutamylcysteine synthetase gene gsh1 and of the ABC transporter gene pgpA in arsenite-resistant Leishmania tarentolae. EMBO J 16, 3057–3065.

14.Tisa, L.S., Rosen, B.P., 1990. Molecular characterization of an anion pump. The ArsB protein is the membrane anchor for the ArsA protein. J Biol Chem 265, 190–194.

15.Lin, Y.-F., Walmsley, A.R., Rosen, B.P., 2006. An arsenic metallochaperone for an arsenic detoxification pump. Proceedings of the National Academy of Sciences of the United States of America 103, 15617–15622.

16.Bobrowicz, P., Wysocki, R., Owsianik, G., Goffeau, A., Ułaszewski, S., 1997. Isolation of three contiguous genes, ACR1, ACR2 and ACR3, involved in resistance to arsenic compounds in the yeast Saccharomyces cerevisiae. Yeast 13, 819–828.

17.Chen, J., Madegowda, M., Bhattacharjee, H., Rosen, B.P., 2015b. ArsP: a methylarsenite efflux permease. Mol Microbiol 98, 625–635.

18.Chen, J., Yoshinaga, M., Garbinski, L.D., Rosen, B.P., 2016. Synergistic interaction of glyceraldehydes-3-phosphate dehydrogenase and ArsJ, a novel organoarsenical efflux permease, confers arsenate resistance. Mol Microbiol 100, 945–953.

1. *Corresponding Authors, E-mail: swj225@126.com [↑](#footnote-ref-1)
